# Supplementary material for: Congenital diaphragmatic hernia in a middle-income country: Persistent high lethality during a 12-year period
Source: PLoS One. 2023 Feb 10;18(2):e0281723. doi: 10.1371/journal.pone.0281723 (PMC9916629; doi:10.1371/journal.pone.0281723)
Supplement: S1 Fig — (PDF) [file pone.0281723.s001.pdf]

Supplementary figure 1. Age in hours at the time of CDH-associated neonatal deaths, according to Kaplan-Meier survival estimate.

(A)

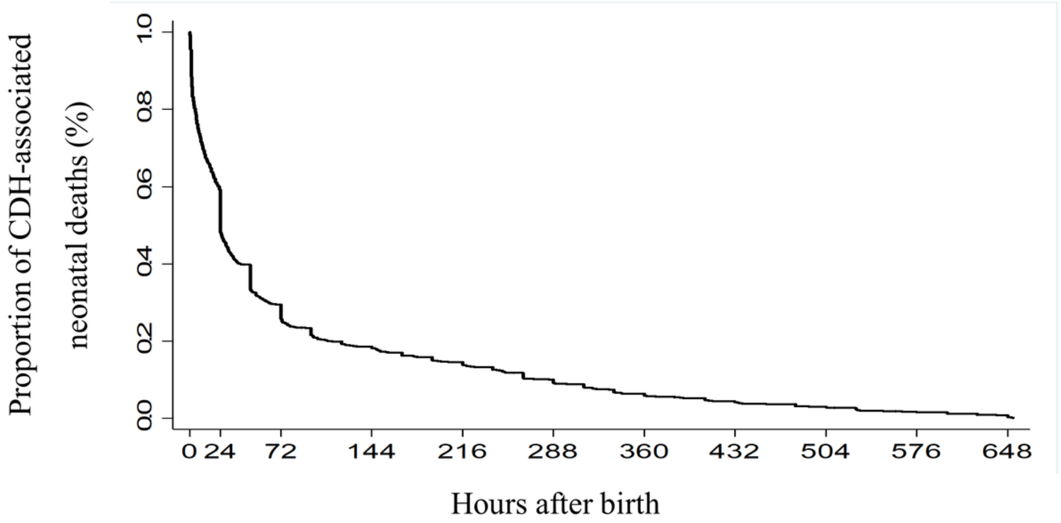

(B)

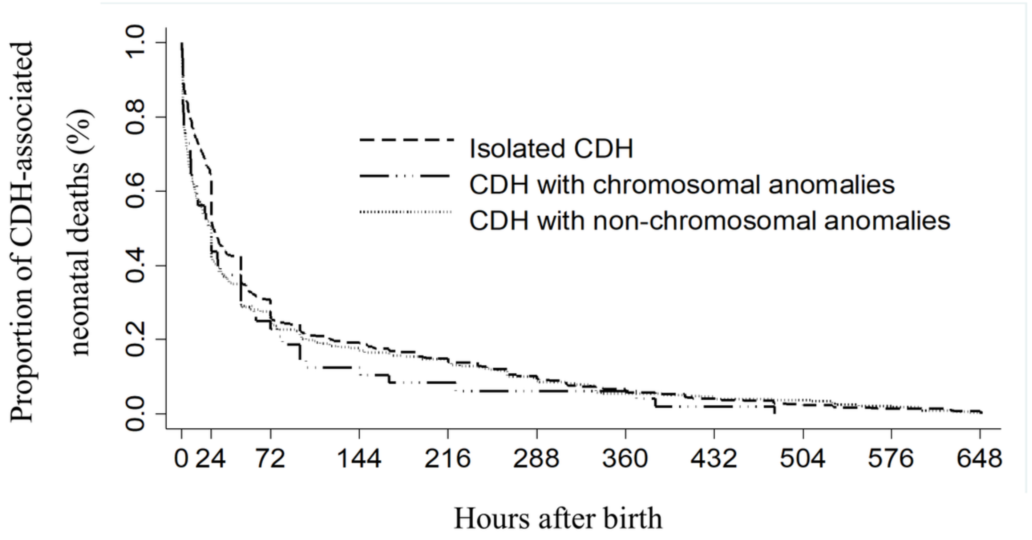

(A) Age in hours at the time of CDH-associated neonatal deaths, according to Kaplan-Meier survival estimate; (B) Age in hours at the time of CDH-associated neonatal deaths for each CDH subgroup, according to Kaplan-Meier survival estimate. CDH: congenital diaphragmatic hernia
