# Supplementary material for: Congenital diaphragmatic hernia in a middle-income country: Persistent high lethality during a 12-year period
Source: PLoS One. 2023 Feb 10;18(2):e0281723. doi: 10.1371/journal.pone.0281723 (PMC9916629; doi:10.1371/journal.pone.0281723)
Supplement: S1 Table — (PDF) [file pone.0281723.s002.pdf]

Supplementary Table 1. Frequency of chromosomal and non-chromosomal anomalies associated with CDH, per diagnoses.

Non-chromosomal congenital anomaly group n=358	n (%)
Nervous system (Q00-Q07)	44 (7.9)
Eye, ear, face and neck (Q10-Q18)	20 (3.6)
Circulatory system (Q20-Q28)	98 (17.7)
Respiratory system (Q30-Q34)	20 (3.6)
Cleft lip and cleft palate (Q35-Q37)	20 (3.6)
Digestive system (Q38-Q45)	24 (4.3)
Genital organs (Q50-Q56)	23 (4.1)
Urinary system (Q60-Q64)	22 (3.9)
Musculoskeletal system (Q65-Q79)	124 (22.3)
Other (Q80-Q89)	160 (28.8)
**Chromosomal anomaly group n=49**	**n (%)**
Edwards Syndrome	23 (47.0)
Patau Syndrome	10 (20.4)
Down Syndrome	3 (6.2)
Other trisomy	5 (10.2)
Other chromosomal anomalies	8 (16.3)
