# Supplementary material for: Congenital diaphragmatic hernia in a middle-income country: Persistent high lethality during a 12-year period
Source: PLoS One. 2023 Feb 10;18(2):e0281723. doi: 10.1371/journal.pone.0281723 (PMC9916629; doi:10.1371/journal.pone.0281723)
Supplement: S2 Table — (PDF) [file pone.0281723.s003.pdf]

Supplementary Table 2. Number of live births, CDH live births and CDH-associated neonatal deaths, per year.

	2004	2005	2006	2007	2008	2009	2010	2011	2012	2013	2014	2015
Live births	599,468	618,898	603,794	595,064	601,637	598,668	601,348	610,185	616,730	611,000	625,333	632,132
CDH live births	91	96	92	77	99	102	109	99	111	110	117	122
CDH-associated neonatal death	77	79	74	64	76	73	83	83	79	80	92	105
Isolated CDH	53	69	62	58	68	69	78	69	68	68	71	85
Isolated CDH-associated neonatal death	39	52	44	47	46	44	54	55	42	45	51	72
CDH with non-chromosomal anomaly	32	23	27	18	26	31	27	27	34	37	41	35
CDH with non-chromosomal anomaly associated neonatal death	32	23	27	16	25	28	25	25	28	30	36	31
CDH with chromosomal anomaly	6	4	3	1	5	2	4	3	9	5	5	2
CDH with chromosomal anomaly associated neonatal deaths	6	4	3	1	5	1	4	3	9	5	5	2
